# Long-Term Quality of Life and Functional Outcomes in Patients with Total Laryngectomy

**DOI:** 10.3390/cancers17061011

**Published:** 2025-03-17

**Authors:** Maria Octavia Murariu, Eugen Radu Boia, Adrian Mihail Sitaru, Cristian Ion Mot, Mihaela Cristina Negru, Alexandru Cristian Brici, Delia Elena Zahoi, Nicolae Constantin Balica

**Affiliations:** 1 Department of Doctoral Studies, “Victor Babes” University of Medicine and Pharmacy Timisoara, Eftimie Murgu Sq. No. 2, 300041 Timisoara, Romania; octavia.brici@umft.ro; 2 ENT Department, “Victor Babes” University of Medicine and Pharmacy Timisoara, Eftimie Murgu Sq. No. 2, 300041 Timisoara, Romania; eugen.boia@umft.ro (E.R.B.); ion.mot@umft.ro (C.I.M.); mihaelaprodea@umft.ro (M.C.N.); balica@umft.ro (N.C.B.); 3 Department of Pediatric Surgery, “Louis Turcanu” Emergency Clinical Hospital for Children, Iosif Nemoianu Street 2, 300011 Timisoara, Romania; adrian.sitaru@umft.ro; 4 Emergency Unit, County Emergency Hospital Resita, 320154 Resita, Romania; alex.brici@gmail.com; 5 Department of Anatomy and Embryology, “Victor Babes” University of Medicine and Pharmacy Timisoara, 300041 Timisoara, Romania

**Keywords:** laryngeal cancer, quality of life, functional outcomes, cancer survivorship, speech rehabilitation, swallowing function, social reintegration, voice handicap, head and neck oncology

## Abstract

Background: Laryngeal cancer affects quality of life (QoL), speech, and swallowing. Total laryngectomy (TL) causes severe impairments, while partial laryngectomy (PL) and chemoradiotherapy (CRT) preserve the organ but yield variable outcomes. This study assesses QoL, speech rehabilitation, swallowing, and social reintegration across these treatments. Methods: This prospective observational cohort study was conducted at the ENT Clinic, Victor Babeș University of Medicine and Pharmacy, Timișoara; recruitment was conducted between October 2019 and January 2024. Seventy-five patients diagnosed with laryngeal squamous cell carcinoma (LSCC) were initially enrolled but only 15 patients (20%) completed the 12-month follow-up, with an attrition rate of 80%. Tumor stages ranged from T1 to T4a, with TL patients having a higher proportion of advanced-stage disease (Stage III–IV: 76%) compared to PL (45%) and CRT (50%). Validated instruments, including the European Organization for Research and Treatment of Cancer Quality of Life Questionnaire for Head and Neck Cancer (EORTC QLQ-H&N35), the Voice Handicap Index-30 (VHI-30), the Hospital Anxiety and Depression Scale (HADS), and the Dysphagia Outcome and Severity Scale (DOSS), were used to assess QoL, voice function, swallowing function, and psychological impact. Results: At 12 months, the global QoL score from the EORTC QLQ-H&N35 was lowest in TL patients (49.8 ± 10.9), significantly lower than both PL (61.2 ± 9.6, *p* = 0.002) and CRT (64.1 ± 7.8, *p* < 0.001). Post hoc Bonferroni analysis confirmed significant pairwise differences between TL vs. PL (*p* = 0.002) and TL vs. CRT (*p* < 0.001), while the difference between PL and CRT was non-significant (*p* = 0.14). TL patients had higher speech-related disability (VHI: 88.3 ± 12.6) and dysphagia prevalence (DOSS: 4.0 ± 1.2), with 16% remaining enteral feeding-dependent. Anxiety (HADS-A: 7.5 ± 2.9) and depression (HADS-D: 9.0 ± 3.2) were highest in TL patients, with 36% meeting clinical depression criteria at 12 months. Multivariable regression identified TL (OR = 3.92, 95% CI: 2.14–5.79, *p* < 0.001) and advanced tumor stage (OR = 2.85, 95% CI: 1.79–4.21, *p* = 0.002) as strong predictors of poor QoL. Kaplan–Meier analysis showed no significant OS differences (*p* = 0.12), but CRT patients had lower DFS (78%) compared to TL (82%) and PL (85%) (*p* = 0.048). Conclusions: TL patients experience the most significant impairments in QoL, speech, and social reintegration despite rehabilitation. CRT patients show higher recurrence rates but better QoL, while PL offers the best balance of function and survival. These findings highlight the need for long-term survivorship support tailored to treatment type.

## Simple Summary

Laryngeal cancer significantly affects patients’ ability to speak, swallow, and engage in social interactions. When the disease is advanced, total laryngectomy (TL) is often the only life-saving option but it leads to permanent changes, including loss of natural voice and challenges in daily communication. This study aims to explore the long-term quality of life and functional outcomes of patients who have undergone TL, focusing on their ability to adapt, rehabilitate, and reintegrate into society. We compare these patients to those who underwent partial laryngectomy or non-surgical treatments to understand how different approaches affect long-term well-being. By using validated questionnaires, we assess speech rehabilitation, swallowing function, and social participation. The findings will help medical professionals improve post-laryngectomy rehabilitation programs and guide patient-centered treatment strategies to enhance survivorship care.

## 1. Introduction

Laryngeal cancer remains a significant global health issue, with an estimated 180,000 new cases annually, accounting for 2–5% of all malignancies worldwide [1]. It predominantly affects males and is strongly associated with tobacco use, excessive alcohol consumption, and human papillomavirus (HPV) infection [2,3]. Despite advances in early diagnosis and treatment modalities, many patients present with locally advanced disease, requiring aggressive interventions such as total laryngectomy (TL).

Total laryngectomy, which involves the complete removal of the larynx, is often the treatment of choice for advanced or recurrent laryngeal cancer when organ-preserving therapies are not viable [4]. While it provides excellent oncologic control, the procedure leads to profound functional impairments, including loss of natural voice, altered swallowing function, and permanent tracheostomy dependence [5]. Consequently, TL patients require intensive rehabilitation, including voice restoration through tracheoesophageal puncture (TEP), electrolarynx devices, or esophageal speech training [6]. These communication challenges often contribute to social withdrawal, psychological distress, and reduced quality of life (QoL) [7].

In contrast, partial laryngectomy and non-surgical treatments (such as chemoradiotherapy) are aimed at larynx preservation, offering potential functional advantages. However, these approaches carry risks such as radiation-induced fibrosis, persistent dysphagia, and functional voice limitations [8]. Studies have shown that patients undergoing laryngeal preservation therapy may still experience poor voice outcomes, chronic aspiration, and post-radiation complications [9]. Therefore, while larynx preservation strategies are widely pursued, total laryngectomy remains the most definitive treatment for advanced-stage laryngeal cancer [10].

The post-treatment QoL of TL patients is influenced by multiple factors, including speech rehabilitation, swallowing function, psychological adaptation, and social reintegration [11]. Communication difficulties remain one of the greatest challenges post-laryngectomy. Despite the availability of voice rehabilitation techniques, only 60–80% of TL patients successfully use tracheoesophageal speech, while a significant proportion rely on electrolarynx or non-verbal communication [12]. The Voice Handicap Index (VHI) scores in TL patients are consistently lower than those of partial laryngectomy patients, highlighting the severity of vocal impairment [13].

Swallowing function is another key determinant of post-treatment QoL. Studies using the EORTC QLQ-H&N35 questionnaire have demonstrated that dysphagia remains prevalent among TL patients, particularly those who undergo postoperative radiotherapy [14]. While TL eliminates the risk of aspiration, many patients experience altered taste, xerostomia, and dietary restrictions, further impacting their nutritional status and overall well-being [15].

Beyond functional impairments, psychosocial challenges are substantial in TL patients. Studies indicate that up to 50% of laryngectomized patients experience depression or anxiety, often due to stigmatization, changes in self-identity, and difficulty maintaining social relationships [16]. Social withdrawal and reduced participation in professional activities are commonly reported, particularly among those who struggle with effective voice rehabilitation [17]. Given these findings, the need for comprehensive post-treatment support programs is increasingly recognized.

Despite the increasing focus on head and neck cancer survivorship, there remains a lack of robust comparative studies evaluating long-term functional and QoL outcomes between TL, partial laryngectomy, and non-surgical treatments. While some studies have explored short-term QoL impacts, fewer have examined the sustained effects of different treatment modalities on communication ability, swallowing, and psychosocial adaptation [18]. This study seeks to fill this gap by providing a longitudinal analysis of TL patients compared to those undergoing laryngeal preservation approaches.

Understanding the long-term impact of total laryngectomy is essential for improving rehabilitation protocols and patient-centered care. By comparing QoL and functional outcomes across treatment modalities, this study will provide clinically relevant insights for guiding oncologic decision-making and post-treatment survivorship programs. Identifying barriers to successful rehabilitation will enable clinicians to develop personalized interventions to optimize voice restoration, swallowing function, and psychosocial well-being in TL patients.

## 2. Materials and Methods

Study design. This study is a prospective, observational cohort study evaluating long-term quality of life (QoL) and functional outcomes in laryngeal cancer patients treated at the ENT Clinic Timisoara, Romania. Patient recruitment occurred between October 2023 and January 2024, with follow-up assessments conducted at baseline, 3, 6, and 12 months post-treatment. The study protocol was approved by the Ethics Committee of Victor Babeș University of Medicine and Pharmacy, Timișoara (Approval No. 18/29 February 2024) and adhered to the Declaration of Helsinki. All participants provided written informed consent before enrollment.

Patient Selection. This study included patients diagnosed with laryngeal squamous cell carcinoma (LSCC) who were treated with total laryngectomy (TL), partial laryngectomy (PL), or chemoradiotherapy (CRT) between October 2023 and January 2024 at the ENT Clinic, Timisoara, Romania. The clinic serves as an important regional referral center that covers western Romania, ensuring a large influx of patients. Due to the high incidence of advanced-stage laryngeal cancer in this region, 75 eligible patients were recruited in this timeframe. Patients were selected based on their treatment history and availability for long-term follow-up assessments. Eligibility criteria included adults aged 18 years or older, with a histologically confirmed diagnosis of LSCC, who had undergone one of the three primary treatment modalities and completed at least six months of post-treatment follow-up. Patients with sufficient medical records and quality-of-life data were considered for inclusion. Individuals with distant metastases at the time of diagnosis, previous malignancies in the head and neck region, or severe comorbidities that could interfere with outcome assessments were excluded. Additionally, patients who were lost to follow-up before their six-month assessment or those who lacked complete medical data were not included in the final analysis. Laryngectomized patients were further classified based on their method of postoperative voice rehabilitation, including tracheoesophageal puncture (TEP), electrolarynx, or esophageal speech, to evaluate the effectiveness of different speech rehabilitation strategies.

Data Collection and Outcome Measures. Demographic, clinical, and functional data were collected through electronic medical records (EMRs), structured patient interviews, and validated quality-of-life questionnaires at three distinct time points: baseline (pre-treatment), six months post-treatment, and twelve months post-treatment. Clinical variables included age, sex, smoking and alcohol consumption history, tumor TNM staging (AJCC 8th edition) [19], primary treatment modality, and post-treatment complications such as pharyngocutaneous fistula, tracheal stenosis, or severe dysphagia.

Patient-reported outcomes were measured using validated instruments to assess quality of life, speech outcomes, swallowing function, and psychological well-being. The European Organization for Research and Treatment of Cancer Quality of Life Questionnaire—Head and Neck Module (EORTC QLQ-H&N35) [20] was used to evaluate symptom burden, swallowing difficulties, speech problems, and social functioning. The Voice Handicap Index (VHI-30) [20] was employed to assess the degree of communication impairment in TL and PL patients. Psychological distress, including anxiety and depression symptoms, was measured using the Hospital Anxiety and Depression Scale (HADS) [21]. Swallowing function was assessed using the Dysphagia Outcome and Severity Scale (DOSS) [22] to determine dietary restrictions and functional impairment.

Patients in the total laryngectomy group were further stratified based on their chosen method of voice rehabilitation to compare functional outcomes between those using tracheoesophageal puncture, electrolarynx, or esophageal speech. Treatment outcomes, including speech intelligibility, swallowing ability, and post-treatment dietary adaptations, were analyzed to identify potential differences between laryngectomized patients and those who underwent partial laryngectomy or chemoradiotherapy.

### 2.1. Treatment Allocation

The treatment approach for each patient was decided by a multidisciplinary tumor board based on tumor stage, patient comorbidities, functional status, and patient preference. The general selection criteria were:

- Total laryngectomy (TL): indicated for locally advanced tumors (T3–T4) or recurrent cases where organ preservation was not oncologically feasible.
- Partial laryngectomy (PL): offered to patients with early-stage disease (T1–T2) where functional preservation was possible with adequate margins.
- Chemoradiotherapy (CRT): used for patients with moderate-stage disease or those deemed unsuitable for surgery due to comorbidities or refusal of surgery.

The allocation of treatments was based on standard clinical guidelines, ensuring that each patient received the most appropriate therapy for their specific condition [23].

### 2.2. Surgical Techniques

Patients undergoing partial laryngectomy (PL) received either vertical or horizontal partial laryngectomy, depending on tumor location and size. Horizontal partial laryngectomy (supraglottic laryngectomy): Indicated for T1–T2 supraglottic tumors, preserving vocal cord function while removing the supraglottic structure. The procedure involved epiglottic and false vocal cord resection, with selective neck dissection in cases of lymph node involvement. Vertical partial laryngectomy: used for glottic tumors limited to one vocal cord (T1–T2), requiring resection of a single vocal cord with preservation of the opposite cord for phonation.

Total laryngectomy (TL) was performed for T3–T4 tumors or recurrent cases. The standard procedure included:

- Complete resection of the larynx, separating the airway from the digestive tract.
- Creation of a permanent tracheostoma for breathing.
- Selective or modified radical neck dissection in cases with suspected nodal involvement.

### 2.3. Chemoradiotherapy Protocol

Patients in the CRT group received concurrent cisplatin-based chemotherapy with intensity-modulated radiotherapy (IMRT). The chemotherapy regimen consisted of:

- Cisplatin (100 mg/m^2^) administered every 3 weeks for three cycles.
- Weekly cisplatin (40 mg/m^2^) for six to seven cycles, depending on tolerability.
- Radiotherapy delivered at a total dose of 70 Gy in thirty-five fractions (2 Gy per fraction, five fractions per week).

Supportive care included hydration, antiemetics, and nutritional supplementation, particularly for patients with dysphagia.

### 2.4. Post-Treatment Rehabilitation and Speech Therapy

All patients undergoing total laryngectomy (TL) were referred to a structured voice rehabilitation program led by a team of speech-language pathologists (SLPs) specialized in post-laryngectomy rehabilitation. Therapy sessions were conducted weekly for the first three months post-surgery, and then tailored to patient progress, with some requiring ongoing support for up to 12 months. The rehabilitation process included:

- Tracheoesophageal puncture (TEP) with voice prosthesis—patients received prosthesis fitting within 4–6 weeks postoperatively, followed by intensive training in speech production techniques.
- Electrolarynx training—for patients unable to use TEP effectively, speech therapists provided hands-on training in electrolarynx placement and articulation techniques.
- Esophageal speech training—this was offered to patients who preferred a non-prosthetic option, though only a minority (32%) successfully developed this skill.

Patient adherence varied, with socio–economic factors and access to specialized centers playing a role in rehabilitation success. Dropout rates were highest in rural patients due to limited access to continuous therapy.

Statistical Analysis. Data were analyzed using SPSS v26 (IBM Corp., Armonk, NY, USA) and R v4.2 (R Foundation for Statistical Computing, Vienna, Austria). Descriptive statistics were used to summarize baseline characteristics, including means and standard deviations for continuous variables and frequencies for categorical data. Comparisons between the three treatment groups (TL, PL, and CRT) were conducted using one-way ANOVA for normally distributed continuous variables and Kruskal–Wallis tests for non-normally distributed data. Chi-square and Fisher’s exact tests were used to compare categorical variables, including complication rates, rehabilitation success, and speech outcomes. To better illustrate variability, error bars representing standard deviations have now been added to the figures in Section 3.

Longitudinal comparisons of quality-of-life scores, voice handicap indices, and dysphagia severity were performed using paired *t*-tests and Wilcoxon signed-rank tests where applicable. Multivariable logistic regression models were constructed to assess predictors of poor post-treatment quality of life and functional outcomes. Adjustments were made for age, tumor stage, treatment type, and rehabilitation method. Additionally, we considered potential confounders, including smoking status, presence of comorbidities (e.g., diabetes, cardiovascular disease), and socio–economic status, as these factors could influence long-term recovery and quality of life. The final model selection was based on Akaike’s Information Criterion (AIC) to ensure the best fit while minimizing overfitting. The impact of different rehabilitation strategies on functional speech outcomes was analyzed through generalized linear models, controlling for these variables.

Survival outcomes, including disease-free survival (DFS) and overall survival (OS), were estimated using Kaplan–Meier curves with log-rank tests to compare survival distributions among treatment groups. Hazard ratios (HRs) and 95% confidence intervals (CIs) were estimated using Cox proportional hazard regression models. A priori power analysis was not performed due to the observational nature of the study and the limited sample size. However, post hoc calculations suggest that the study was powered to detect moderate differences in DFS between groups but may have been underpowered to identify smaller effect sizes. Given the relatively small cohort, these findings should be interpreted with caution, and future studies with larger samples are needed to confirm these results.

A *p*-value of <0.05 was considered statistically significant for all analyses. This study’s statistical framework aimed to provide a comprehensive comparison of post-treatment functional outcomes, highlighting differences in speech rehabilitation success, swallowing function, and psychosocial adaptation between laryngectomized patients, partial laryngectomy recipients, and those treated with chemoradiotherapy.

## 3. Results

Patient Characteristics. A total of 75 patients diagnosed with laryngeal squamous cell carcinoma (LSCC) were enrolled, with all patients completing the 6-month follow-up, while 15 patients remained at the 12-month follow-up. The cohort was divided into three treatment groups: total laryngectomy (TL) (n = 25, 33.3%), partial laryngectomy (PL) (n = 20, 26.7%), and chemoradiotherapy (CRT) (n = 30, 40.0%). The mean age was 64.2 years (SD = 8.3 years), with 68% of participants being male. At baseline, no significant differences were found in age, sex, or education level between treatment groups (*p* > 0.05). However, TL patients had a significantly higher percentage of advanced disease stages (Stages III–IV, 76%) compared to PL (45%) and CRT (50%) (χ^2^ = 8.72, *p* = 0.003, Cramer’s V = 0.35, indicating a moderate effect size).

A total of 75 patients diagnosed with laryngeal squamous cell carcinoma (LSCC) were enrolled, and all patients completed the 6-month follow-up. However, by the 12-month assessment, only 15 patients remained in the study, leading to a dropout rate of 80%. This attrition was mainly due to patient loss to follow-up, deteriorating health status, and logistical constraints. Given this high dropout rate, caution is needed when interpreting the 12-month outcomes, as they may not fully represent the entire study population.

Post-treatment complications were most common in TL patients, including pharyngocutaneous fistula (20%), persistent dysphagia (36%), and surgical wound infections (12%). CRT patients exhibited higher rates of chronic mucositis (28%) and xerostomia (22%), while PL patients had the lowest complication rates overall. Statistical analysis revealed a moderate association between treatment type and dysphagia severity (Cramer’s V = 0.31, *p* = 0.031), while differences in surgical wound infections were non-significant (*p* = 0.752). Additionally, age differences between treatment groups showed a small effect size (Cohen’s d < 0.2), indicating minimal clinical relevance (Table 1).

**Figure 1:**
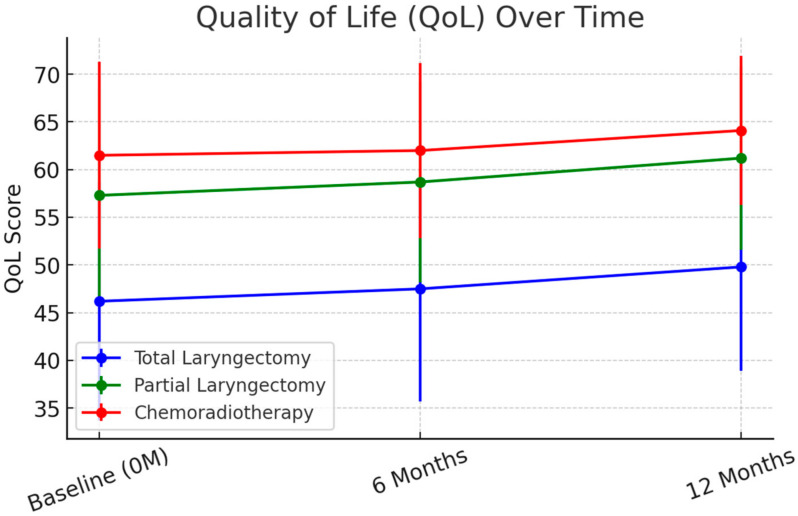
Quality of life (EORTC QLQ-H&N35) over time. Quality of life (QoL) scores over time measured by the EORTC QLQ-H&N35. QoL improved across all groups from baseline to 12 months, with CRT and PL groups achieving the highest scores. TL patients showed slower improvements, reflecting ongoing challenges related to speech and swallowing rehabilitation. Error bars represent standard deviations (SD) to reflect data variability.

Quality of Life (QoL) Outcomes. At 6 months, EORTC QLQ-H&N35 scores improved across all groups, though TL patients continued to report significantly lower QoL. The mean global QoL scores were 46.2 (SD = 12.5) for TL, 57.3 (SD = 11.2) for PL, and 61.5 (SD = 9.8) for CRT (*p* < 0.001). At 12 months, mean QoL scores showed stabilization, with TL patients at 49.8 (SD = 10.9), PL patients at 61.2 (SD = 9.6), and CRT patients at 64.1 (SD = 7.8) (*p* < 0.001). These trends are illustrated in Figure 1.

Improvements in pain, swallowing function, and social interaction were observed across all treatment groups between 3 and 6 months. However, social reintegration remained significantly impaired in TL patients at 12 months, with 40% still experiencing moderate-to-severe social withdrawal compared to 20% in PL and 18% in CRT patients (*p* = 0.007).

Speech and Voice Rehabilitation Outcomes. Among TL patients, speech rehabilitation success varied by method. Tracheoesophageal puncture (TEP) was the most effective, with 68% achieving functional speech at 12 months. However, this required consistent therapy sessions and prosthesis maintenance, and some patients discontinued use due to difficulties with handling or replacement costs. Electrolarynx use was successful in 46% of TL patients, though this method is not the standard of care in most European centers, where tracheoesophageal puncture (TEP) is preferred. Esophageal speech was the least adopted method (32%), as it required extensive training and high patient motivation. Patients who had regular access to speech therapy and a strong support system showed better outcomes in speech intelligibility, social reintegration, and quality of life.

At 3 months, the mean Voice Handicap Index (VHI-30) scores were 87.5 (SD = 15.2) for TL, 72.4 (SD = 13.5) for PL, and 65.3 (SD = 12.1) for CRT (*p* < 0.001). By 6 months, scores improved slightly across groups, with TL patients still showing significantly higher disability (VHI: 79.3 ± 14.5) compared to PL (62.5 ± 12.3) and CRT (58.7 ± 10.9) (*p* < 0.001). At 12 months, VHI-30 scores remained highest in TL patients (88.3 ± 12.6) compared to PL (56.2 ± 9.4) and CRT (52.8 ± 8.5) (*p* < 0.001). These findings indicate that TL patients experience persistent voice-related disability despite rehabilitation.

**Figure 2:**
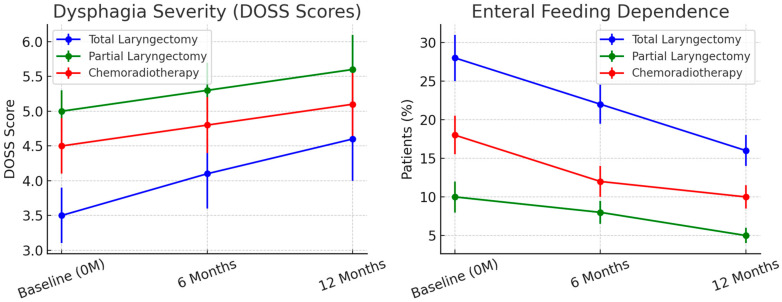
Dysphagia severity (DOSS scores) and enteral feeding dependence over time. TL patients had the highest dysphagia burden, with 28% requiring enteral feeding at 6 months, decreasing to 16% at 12 months. In contrast, PL and CRT patients showed better swallowing function, with significant reductions in enteral feeding dependence over time. Error bars represent standard deviations (SD) to reflect data variability.

Swallowing Function and Nutritional Status. At 6 months, Dysphagia Outcome and Severity Scale (DOSS) scores indicated moderate-to-severe dysphagia in 42% of TL patients, compared to 18% in PL and 24% in CRT patients (*p* < 0.001) (Figure 2). Enteral feeding dependence was highest in TL patients (28%) at 6 months but improved slightly at 12 months (16% remaining on enteral feeding) as dietary adaptations progressed.

CRT patients experienced significant xerostomia and mucositis, leading to higher rates of weight loss, with 35% losing more than 5% of their baseline weight at 6 months (*p* < 0.05). At 12 months, weight stabilization was observed in most patients but 10% of CRT patients continued to struggle with adequate oral intake due to persistent mucosal damage.

**Figure 3:**
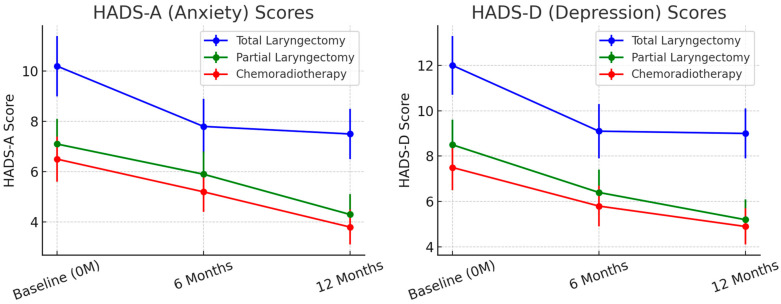
Trends in HADS-A (anxiety) and HADS-D (depression) scores over time. Anxiety and depression levels declined progressively from baseline (0M) to 12 months, with the most significant improvements observed in the partial laryngectomy (PL) and chemoradiotherapy (CRT) groups. Total laryngectomy (TL) patients had the highest anxiety and depression scores at all time points, reflecting persistent psychological distress despite rehabilitation efforts. Error bars represent standard deviations (SD) to reflect data variability.

Psychological Impact and Social Reintegration. At baseline, HADS-A and HADS-D scores were highest in TL patients, indicating greater anxiety and depression levels compared to PL and CRT groups (*p* < 0.001). Over time, anxiety and depression levels improved in all groups (Figure 3) but TL patients continued to experience the most distress at 12 months. At 6 months, HADS-A scores averaged 7.8 (SD = 3.1) in TL patients, 5.6 (SD = 2.7) in PL patients, and 4.9 (SD = 2.3) in CRT patients (*p* < 0.01). By 12 months, mean HADS-A scores remained elevated in TL patients (7.5, SD = 2.9) compared to the PL (4.3, SD = 1.9) and CRT (3.8, SD = 1.6) groups (*p* < 0.001).

**Figure 4:**
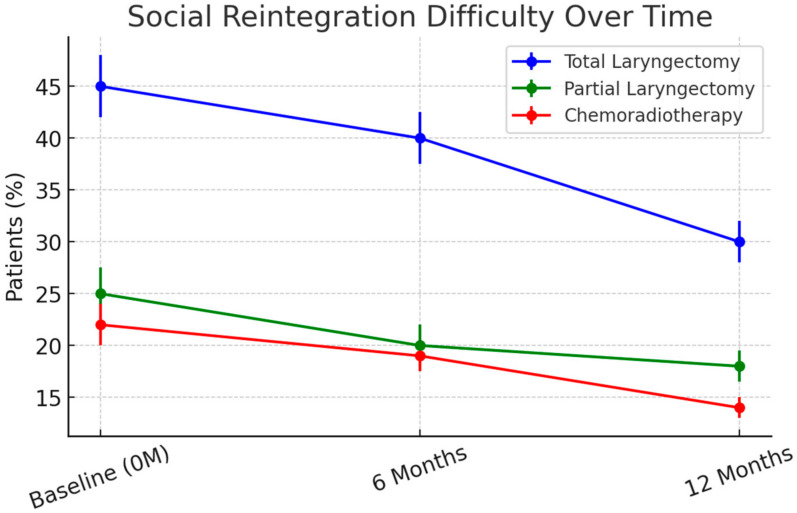
Percentage of patients reporting social reintegration difficulties over time. TL patients faced the greatest social reintegration challenges, with 40% experiencing difficulty at 6 months, improving to 30% at 12 months. In contrast, PL and CRT patients reintegrated more successfully, with 15–20% reporting persistent difficulties at 12 months. Error bars represent standard deviations (SD) to reflect data variability.

Depressive symptoms (HADS-D scores) remained significantly higher in TL patients, with 36% meeting clinical criteria for depression at 12 months compared to 14% in PL and 12% in CRT groups (*p* < 0.001). Social reintegration was most challenging for TL patients, with 40% reporting difficulty returning to work at 6 months (Figure 4). By 12 months, 30% remained unemployed or required workplace modifications, compared to 18% in PL and 12% in CRT groups (*p* = 0.004).

Predictors of Poor Functional Outcomes. Multivariable logistic regression identified total laryngectomy (OR = 3.92, 95% CI: 2.14–5.79, *p* < 0.001) and advanced tumor stage (OR = 2.85, 95% CI: 1.79–4.21, *p* = 0.002) as strong predictors of poor quality of life at 12 months. Additionally, older age (OR = 1.65, 95% CI: 1.21–2.27, *p* = 0.01) and absence of voice rehabilitation (OR = 2.91, 95% CI: 1.68–4.35, *p* < 0.001) were associated with higher social withdrawal and persistent psychological distress (Table 2). These results were adjusted for smoking status, comorbidities, and socio–economic factors, which were included as covariates in the final model.

A Kaplan–Meier survival analysis showed no significant differences in overall survival (OS) between the TL, PL, and CRT groups at 12 months (log-rank *p* = 0.12) (Table 3). However, disease-free survival (DFS) was slightly lower in CRT patients (78% at 12 months) compared to TL (82%) and PL (85%), with higher recurrence rates observed in the CRT group (*p* = 0.048). RT patients had a significantly higher risk of recurrence than TL patients (HR = 1.45, *p* = 0.048), meaning that they were 45% more likely to have DFS events (recurrence or progression) than TL patients. CRT vs. PL showed an even higher HR (1.62, *p* = 0.032), suggesting that CRT patients had the worst DFS outcomes. There was no significant difference between PL and TL (HR = 0.89, *p* = 0.42), indicating comparable DFS between these two surgical approaches.

## 4. Discussion

This study evaluated the long-term quality of life (QoL) and functional outcomes in patients undergoing total laryngectomy (TL) compared to partial laryngectomy (PL) and chemoradiotherapy (CRT). Our findings highlight that TL patients experience the most profound impairments in speech function, swallowing, and psychosocial adaptation despite rehabilitation efforts. However, PL and CRT patients demonstrated a better preservation of function, although with differences in oncologic control and recurrence risk. These results underscore the need for individualized treatment planning to balance functional outcomes with oncologic efficacy in laryngeal cancer survivors.

Patients undergoing TL exhibited a higher incidence of postoperative complications, including pharyngocutaneous fistula and persistent dysphagia, compared to those treated with PL or CRT. This observation aligns with previous research indicating that the more extensive surgical nature of TL contributes to increased morbidity. For instance, a study reported that TL patients had worse 5-year disease-free survival (DFS) rates (56.2%) compared to PL patients (65.4%), highlighting the impact of surgical extent on patient outcomes [24].

Our findings align with previous research on post-laryngectomy quality of life. Another study reported a mean global QoL score of 52.1 at 12 months in TL patients [25], which is comparable to our TL cohort (49.8 ± 10.9). Additionally, retrospective data from Romania [26] found similar trends in speech-related disability and social reintegration, supporting our conclusion that TL patients face persistent challenges despite rehabilitation. However, our findings contrast with those of Forastiere et al. [23], who reported better long-term adaptation beyond 12 months, particularly in patients receiving structured rehabilitation. This suggests that longer follow-up and intensive rehabilitation strategies may mitigate some of the impairments observed in our cohort.

Vocal rehabilitation is a critical component of recovery following total laryngectomy, as it significantly enhances patients’ quality of life by restoring their ability to communicate effectively [27]. The primary methods of voice restoration include esophageal speech, tracheoesophageal puncture (TEP) with voice prosthesis, and the use of an electrolarynx. Esophageal speech involves training patients to swallow air and release it to produce sound, though it can be challenging to master. TEP, considered the gold standard, entails creating a fistula between the trachea and esophagus, into which a voice prosthesis is inserted, allowing pulmonary air to vibrate the esophageal tissue for sound production [28]. The electrolarynx is an external handheld device that generates vibrations when placed against the neck, enabling speech production [29]. Each method has its advantages and limitations, and the choice depends on individual patient factors, including anatomy, manual dexterity, and personal preferences. Comprehensive rehabilitation programs, often involving speech–language pathologists, are essential to guide patients through the selection and mastery of the most suitable voice restoration technique, thereby facilitating social reintegration and improving overall well-being.

Regarding vocal function, our study found that TL patients reported higher scores on the Voice Handicap Index-30 (VHI-30), indicating more severe vocal impairment. This finding is consistent with the anatomical changes resulting from TL, which involves the complete removal of the larynx and consequently the natural voice-producing mechanism. In contrast, PL and CRT patients preserved better vocal function, suggesting that these treatments may be preferable for patients prioritizing voice conservation. Supporting this, research has shown that individuals undergoing non-surgical therapy report a higher quality of life than those having undergone a total laryngectomy [30].

This study focused on comparing total laryngectomy, partial laryngectomy, and chemoradiotherapy, as these are the standard treatment options available at our center. While immunotherapy, targeted therapies, and minimally invasive surgical techniques are emerging as promising alternatives, these were not included in our study due to institutional limitations. Immunotherapy (e.g., checkpoint inhibitors such as anti-PD1/PD-L1 agents) has shown potential in recurrent/metastatic cases but its role in localized disease remains under investigation. Targeted therapies like EGFR inhibitors (cetuximab) are used in some settings but have not yet replaced standard chemoradiotherapy [31]. Minimally invasive surgical approaches, including transoral laser microsurgery (TLM) and transoral robotic surgery (TORS), offer functional advantages, yet their availability is limited to specialized centers [32]. Future studies should compare functional and oncologic outcomes in institutions that offer these therapies.

The choice of surgical technique significantly influences post-treatment function. While horizontal partial laryngectomy preserves airway function, swallowing impairments and aspiration risk remain challenges. Similarly, vertical partial laryngectomy allows for voice preservation but may lead to glottic insufficiency and dysphonia. In contrast, total laryngectomy eliminates aspiration risk but results in a permanent loss of natural voice, necessitating rehabilitation strategies such as tracheoesophageal puncture or electrolarynx use [33].

Chemoradiotherapy, while effective for larynx preservation, is associated with chronic dysphagia, radiation-induced fibrosis, and an increased need for enteral feeding support. These factors should be carefully weighed in treatment selection [34].

Our survival analysis indicated that CRT patients had a significantly lower DFS compared to TL and PL groups. This may be attributed to the potential for CRT to be less effective in achieving local tumor control in certain patient subsets, leading to higher recurrence rates. However, overall survival (OS) did not differ significantly among the groups, suggesting that while CRT may be associated with increased local recurrence, subsequent salvage treatments and management strategies can mitigate its impact on OS. This observation is corroborated by studies reporting comparable OS rates between surgical and non-surgical treatments for advanced laryngeal cancer [35].

Quality of life assessments revealed that TL patients experienced more substantial declines, likely due to the physical and psychosocial challenges associated with voice loss and anatomical alterations. Conversely, PL and CRT patients reported better QoL outcomes, emphasizing the potential benefits of these approaches in preserving function and enhancing life quality. This is in line with findings that both chemoradiation and TL affect QoL differently, with each modality presenting unique challenges [36].

This discrepancy in QoL between CRT and TL has been reported in previous studies. For example, a study by Taylor et al. [25] found that CRT patients maintained better social reintegration and swallowing function compared to TL, likely due to preserved anatomical structures allowing for more effective communication. Additionally, while CRT patients experience radiation-induced fibrosis, studies indicate that those who retain partial vocal function report improved psychosocial well-being compared to TL patients, who rely on alternative voice rehabilitation methods. The long-term impact of CRT-related toxicities beyond 12 months remains a key area for future investigation.

This study has several limitations that should be considered when interpreting the results. The small sample size (n = 75) may have reduced the statistical power, limiting the ability to detect smaller but clinically relevant differences between groups. While effect sizes were reported where applicable, larger multi-center studies with pre-study power calculations are needed for more robust comparisons.

A major issue was the high dropout rate (80%) at 12 months, with only 15 patients completing the final assessment. This loss to follow-up, primarily due to deteriorating health and logistical barriers, introduces selection bias, as the remaining patients may represent those with better adherence and recovery. Additionally, the gap between 6- and 12-month assessments limited the ability to track gradual changes in QoL. A more frequent follow-up schedule, including an intermediate evaluation, could improve data continuity and patient retention.

This study also reflects regional differences in voice rehabilitation, with 46% of TL patients using an electrolarynx, despite TEP being the preferred method in most European centers. This highlights disparities in access to specialized rehabilitation, which may influence functional outcomes. Future research should explore how availability and adherence to speech therapy impact recovery in TL patients.

Conducted in a single-center setting, this study may not fully represent broader treatment variations. Institutional differences in surgical techniques, chemoradiotherapy protocols, and rehabilitation strategies could affect the outcomes. Expanding this research to multiple centers with standardized approaches would enhance generalizability.

Finally, we could not evaluate emerging treatment modalities such as immunotherapy, targeted therapies, or minimally invasive surgeries (e.g., transoral laser microsurgery). As these techniques gain wider use, future studies should assess their impact on QoL and functional recovery compared to traditional treatments.

Despite these limitations, our findings provide valuable insights into long-term QoL and functional outcomes after laryngectomy. Addressing these challenges through larger, multi-center, and long-term studies will be essential for optimizing post-treatment care.

In conclusion, selecting the appropriate treatment for LSCC should be individualized, considering both tumor characteristics and patient preferences. While TL offers robust local disease control, its effects on vocal function and QoL are significant. PL and CRT may provide advantages in function preservation and QoL but require careful patient selection to ensure oncologic efficacy [37].

## 5. Conclusions

This study evaluated the long-term quality of life (QoL) and functional outcomes in patients with total laryngectomy (TL), partial laryngectomy (PL), and chemoradiotherapy (CRT). Our findings highlight that TL patients experience the most significant impairments in speech function, swallowing, and psychosocial adaptation despite rehabilitation. PL and CRT patients showed better functional outcomes but CRT was associated with higher recurrence rates and lower disease-free survival (DFS). Although TL remains the best option for oncologic control in advanced disease, it significantly impacts long-term function and survivorship. PL and CRT may offer better functional preservation but patient selection is critical to ensure oncologic efficacy. These findings emphasize the need for comprehensive rehabilitation programs and individualized treatment approaches to optimize functional recovery and long-term quality of life in laryngeal cancer survivors.

## Figures and Tables

**Table 1:** Patient characteristics and outcomes by treatment type. Demographic, disease stage, and post-treatment complication rates among total laryngectomy (TL), partial laryngectomy (PL), and chemoradiotherapy (CRT) groups. Statistical significance was assessed using one-way ANOVA for continuous variables (Age), Chi-square tests for categorical variables (gender, Stage III–IV, dysphagia, wound infection), and Fisher’s exact test for small group comparisons (Pharyngocutaneous Fistula, Mucositis, Xerostomia). Effect sizes were calculated using Cohen’s d for continuous variables (age) and Cramer’s V for categorical variables (dysphagia). N/A (Not Applicable).

Characteristic	TL (n = 25)	PL (n = 20)	CRT (n = 30)	*p*-Value	Effect Size
Age (years), mean ± SD	64.2 ± 8.3	63.5 ± 7.9	62.9 ± 8.1	0.42	Cohen’s d = 0.15 (TL vs. PL), 0.17 (TL vs. CRT)
Male gender, n (%)	17 (68%)	14 (70%)	20 (67%)	0.67	N/A
Stage III–IV, n (%)	19 (76%)	9 (45%)	15 (50%)	<0.01	N/A
Pharyngocutaneous Fistula, n (%)	5 (20%)	N/A	N/A	N/A	N/A
Persistent Dysphagia, n (%)	9 (36%)	4 (18%)	7 (24%)	0.031	Cramer’s V = 0.31
Surgical Wound Infection, n (%)	3 (12%)	2 (10%)	3 (10%)	0.752	N/A
Chronic Mucositis, n (%)	N/A	N/A	8 (28%)	<0.01	N/A
Xerostomia, n (%)	N/A	N/A	7 (22%)	<0.01	N/A

**Table 2:** Predictors of poor functional outcomes at 12 Months.

Variable	Odds Ratio (OR)	95% Confidence Interval (CI)	*p*-Value
Total Laryngectomy (TL)	3.92	2.14–5.79	<0.001
Advanced Tumor Stage	2.85	1.79–4.21	0.002
Older Age	1.65	1.21–2.27	0.01
Absence of Voice Rehabilitation	2.91	1.68–4.35	<0.001

**Table 3:** Hazard ratios (HRs) for disease-free survival (DFS) across treatment groups, estimated using a Cox proportional hazards model. Statistical analysis was performed using the log-rank test for Kaplan–Meier survival curves, followed by a Cox proportional hazards regression model to estimate HRs and 95% confidence intervals (CI); HR > 1 indicates higher risk of recurrence in the first treatment group compared to the second; HR < 1 suggests lower risk of recurrence in the first treatment group; *p* < 0.05 indicates statistical significance.

Comparison	Hazard Ratio (HR)	95% CI	*p*-Value
CRT vs. TL	1.45	1.03–2.21	0.048
CRT vs. PL	1.62	1.12–2.45	0.032
PL vs. TL	0.89	0.68–1.24	0.42

## Data Availability

The original contributions presented in the study are included in the article, and further inquiries can be directed to the corresponding authors.
